# Kommt die Defi-Drohne? Einsatz von Drohnen zur Verbesserung der Frühdefibrillationsrate

**DOI:** 10.1007/s00101-022-01204-w

**Published:** 2022-09-27

**Authors:** Karl-Christian Thies, Gerrit Jansen, Dirk Wähnert

**Affiliations:** 1 Universitätsklinik für Anästhesiologie, Intensiv‑, Notfallmedizin, Transfusionsmedizin und Schmerztherapie, Evangelisches Klinikum Bethel gGmbH, Universitätsklinikum OWL der Universität Bielefeld, Campus Bielefeld-Bethel, Burgsteig 13, 33617 Bielefeld, Deutschland; 2 Klinik für Unfallchirurgie und Orthopädie, Evangelisches Klinikum Bethel gGmbH, Universitätsklinikum OWL der Universität Bielefeld, Campus Bielefeld-Bethel, Burgsteig 13, 33617 Bielefeld, Deutschland

**Keywords:** Unmanned Aerial Device, Herz-Kreislauf-Stillstand, First Responder, Defibrillator, Automatischer externer Defibrillator, Unmanned aerial device, Cardiac arrest, First responder, Defibrillator, Automated external defibrillator

## Abstract

**Hintergrund:**

Der Mangel an automatischen externen Defibrillatoren (AED) und die fehlende Kenntnis von Ersthelfern im Umgang mit diesen Geräten haben in Deutschland zu einer ungenügenden Verbreitung der Public-Access-Defibrillation geführt.

**Fragestellung:**

Dieser Artikel untersucht, inwieweit die automatisierte Zuführung von AED bei außerklinischem Herz-Kreislauf-Stillstand mithilfe von Drohnen hier Abhilfe schaffen kann.

**Material und Methodik:**

Narrative Literaturübersicht, Auswertung von Statistiken, Analyse relevanter Medienmeldungen und Diskussion von Grundlagenarbeiten.

**Ergebnisse:**

Die vorliegenden Untersuchungen sind überwiegend im experimentellen Bereich angesiedelt und belegen die Machbarkeit und die Sicherheit des Drohneneinsatzes sowie eine Verkürzung der Zeit bis zur Erstdefibrillation. Erste klinische Studien bestätigen dies.

**Schlussfolgerung:**

Defi-Drohnen könnten wahrscheinlich zur Verbesserung der Frühdefibrillationrate in Deutschland beitragen. Dies gilt sowohl für den ländlichen als auch den urbanen Raum. Die technologischen Voraussetzungen sind gegeben, die flugrechtlichen Bedingungen müssten allerdings noch angepasst werden. Um das volle Potenzial der neuen Technologie auszuloten, sind weitere Feldversuche erforderlich.

**Video online:**

Die Online-Version dieses Beitrags (10.1007/s00101-022-01204-w) enthält weitere Videos*.*

## Abstract (en)

**Background:**

The poor availability of automatic external defibrillators (AED) and the modest knowledge of lay persons in handling these devices has led to an insufficient spread of public access defibrillation in Germany.

**Objective:**

This article examines whether the automated deployment of AED drones to out-of-hospital cardiac arrest can help to remedy this situation.

**Methods:**

Narrative literature review, evaluation of statistics, analysis of relevant media reports, and discussion of key research.

**Results:**

The present investigations are mainly located in the experimental field and demonstrate the feasibility and safety of drone use, as well as shorter times to first defibrillation, which is confirmed by initial clinical studies. Mathematical models also indicate cost-effectiveness of airborne AED delivery compared to ground dispatch. Integration into the chain of survival appears to be possible but adaptations to existing emergency medical service structures and close cooperation with regional first responder and AED schemes as well as local authorities is required to optimise patient benefit and efficiency.

**Conclusion:**

The use of AED drones could probably contribute to improving public access defibrillation in Germany. This applies to both rural and urban regions. The technological requirements are met but flight regulations still have to be amended. In order to explore the full potential of this novel technology, further field trials are required to achieve smooth integration into existing emergency medical services.

Die Frühdefibrillation sowie der sofortige Beginn der Reanimation durch Umstehende sind der Schlüssel zur erfolgreichen Behandlung des Herz-Kreislauf-Stillstandes. In Deutschland allerdings gelangt die Frühdefibrillation aufgrund eines Mangels öffentlich zugänglicher automatischer externer Defibrillatoren (AED) kaum zur Anwendung. Im Rahmen eines Gesamtkonzepts zur Anhebung der Frühdefibrillationsrate hat das „International Liaison Committee on Resuscitation“ auch den Einsatz von Drohnen empfohlen, um AED schneller an die Einsatzstelle zu bringen [7]. Das Konzept steckt noch in den Kinderschuhen, im Januar 2022 jedoch erregte die erste erfolgreiche Defibrillation mit einem per Drohne zugeführten AED weltweit öffentliche Aufmerksamkeit [15].

## Ersthelferreanimation und Frühdefibrillation – eine Standortbestimmung

Laut Jahresbericht des Reanimationsregisters wurden in Deutschland im Jahr 2020 ca. 60.000 PatientInnen nach außerklinischem Herz-Kreislauf-Stillstand (OHCA) reanimiert. Bei nur 21 % dieser PatientInnen lag bei Eintreffen des Rettungsdienstes ein defibrillierbarer Rhythmus vor. Die Krankenhausentlassungsrate lag bei 10,5 %, die Rate der PatientInnen, die ohne oder mit leichtem neurologischem Defizit entlassen wurden, lag bei nur 6,8 % [17]. Das OHCA-Outcome bleibt ernüchternd und ist trotz aller Bemühungen über die letzten 40 Jahre, auch im internationalen Kontext, mehr oder weniger unverändert niedrig geblieben [34, 35].

### Internationale Flughäfen zeigen, was möglich ist

Eine deutlich bessere Bilanz zeigen die Reanimationsergebnisse internationaler Flughäfen. Die Überlebenswahrscheinlichkeit ist hier 3‑ bis 4‑mal höher als im deutschen Durchschnitt [26, 27]. Die weltweiten besten Zahlen legt Heathrow Airport vor, wo nach eigenen Angaben 74 % aller Patienten mit einem Herz-Kreislauf-Stillstand lebend das Krankenhaus verlassen [22]. Ursächlich für die guten Ergebnisse sind, neben den günstigeren epidemiologischen Voraussetzungen auf Flughäfen, der unmittelbare Beginn der Reanimation durch geschulte Mitarbeiter, sofort erreichbare automatische externe Defibrillatoren (AED) und die schnelle Verfügbarkeit qualifizierter Paramedics [23]. Der stärkste Prädiktor fürs Überleben eines Herz-Kreislauf-Stillstands ist neben dem Vorliegen eines defibrillierbaren Rhythmus die Frühdefibrillation durch Ersthelfer [27], denn innerhalb der ersten 2 min nach einem Herz-Kreislauf-Stillstand liegt die Prävalenz defibrillierbarer Rhythmen über 75 %. Sie fällt dann konsekutiv ab [31].

### Wie sieht’s bei den Nachbarn aus? – Beispiel Niederlande

Ein Blick über unsere Grenzen zeigt allerdings, dass deutlich bessere Reanimationsergebnisse nicht nur an Flughäfen erzielt werden können.

Laut Bericht der niederländischen „Hartstichting“ aus dem Jahr 2016 war im Rettungsdienstbereich „Noord Holland“ im Jahr 2014 bei 65 % aller Patienten (*n* = 891) schon vor Eintreffen des Rettungsdienstes durch Ersthelfer ein AED angeschlossen. Die Prävalenz defibrillierbarer Rhythmen lag bei 47 %. Es wurden 23 % der Reanimierten lebend aus dem Krankenhaus entlassen, wovon 95 % ein gutes bis sehr gutes neurologisches Outcome erreichten (Cerebral Performance Category von 1–2) [21].

In Deutschland liegen die Entlassungsraten nach außerklinischem Herz-Kreislaufstillstand bei nur 11 %, und nur 7 % aller Patienten erreichen eine CPC von 1–2 [16, 17]. Interessanterweise lagen die Eintreffzeiten des niederländischen Rettungsdienstes mit im Mittel 9 min deutlich über den deutschen Werten. Bemerkenswert ist der hohe Verbreitungs- und Organisationsgrad von First-Responder-Systemen in den Niederlanden. Im Jahr 2021 waren 1,5 % der niederländischen Bevölkerung beim landesweiten Alarmierungssystem „Hartslag nu!“ als Ersthelfer registriert. Darüber hinaus sind alle Polizei und Feuerwehrfahrzeuge mit AED ausgestattet und werden von den Rettungsleitstellen eingesetzt, falls sie schneller als der reguläre Rettungsdienst vor Ort sein können [20].

### AED und Ersthelfer – die Kombination macht den Unterschied

Die Integration von Ersthelferreanimation und Frühdefibrillation in die Rettungskette bewirkt also den wesentlichen Unterschied. Krankenhausentlassungsraten von 53 % bzw. 23 % werden beschrieben, wenn Ersthelfer bzw. First Responder früh defibrillieren [2]. Der Zahl der Ersthelferreanimationen in Deutschland hat sich in den letzten Jahren positiv entwickelt; die intensive Öffentlichkeitsarbeit, Telefonreanimation und der Einsatz von „First Respondern“ zeigen zumindest regionale Wirkung [40]. Diese Wirkung bleibt derzeit allerdings begrenzt, da die Frühdefibrillation bei uns so gut wie keine Anwendung findet. Nach Auskunft des Reanimationsregisters liegt in Deutschland die Frühdefibrillationsrate unter 2 %. Dies ist nachvollziehbar, da die Zahl der registrierten AED in den letzten Jahren zwar zugenommen hat, die AED-Dichte mit 0,09/km^2^ [13] aber immer noch weit hinter anderen Ländern zurückliegt (Niederlande 0,6 AED/km^2^) [20]. Das European Resuscitation Council empfiehlt sogar eine Abdeckung von 2 AED/km^2^ [39].

### AED – Nutzung und Verfügbarkeit

Auch ist die allgemeine Kenntnis in der Bevölkerung über den Zweck, die Funktion und Lokalisation von AED laut einer Umfrage der ADAC Stiftung sehr beschränkt, was einer breiten und kosteneffizienten Anwendung durch Ersthelfer entgegensteht. Darüber hinaus besteht bei Standortauswahl, Unterhalt und Zugang zu den aufgestellten AED erheblicher Nachbesserungsbedarf [1, 25]. In reinen Wohngegenden sind AED kaum zu finden und werden dort in der Regel auch nicht genutzt [19]. AED werden in erster Linie an öffentlichen Einrichtungen, wie Behörden, Bahnhöfen und Sportplätzen sowie in Betrieben aufgestellt. Die Verfügbarkeit ist daher abhängig von den Öffnungszeiten dieser Einrichtungen. Dem steht entgegen, dass sich 66 % der Herz-Kreislauf-Stillstände im häuslichen Umfeld ereignen [17] und damit weitab vom nächsten AED-Standort. Einmal aufgestellt, ist auch der Unterhalt der Geräte nicht immer sichergestellt; wer achtet auf die Einhaltung von Wartungsintervallen, wer wechselt abgelaufene Klebeelektroden oder Batterien? Mehr als die Hälfte der aufgestellten Geräte ist Schätzungen zufolge nicht ausreichend gewartet [12].

AED-Register, die von Rettungsleitstellen genutzt werden könnten, um Ersthelfer zum nächsten AED-Standort zu dirigieren, sind erst im Aufbau begriffen und werden nur selten genutzt.

Deutschland ist demnach weit von flächendeckend funktionierenden AED-Netzwerken entfernt. Diese sind allein aus Kostengründen schwer darstellbar, zumal weder Ersthelfersysteme noch Frühdefibrillation zur öffentlichen Daseinsvorsorge gehören und auch nicht im Sozialgesetzbuch V abgebildet sind. Eine konsequente Finanzierung aus öffentlichen Mitteln oder Krankenkassenbeiträgen ist daher, trotz des nachgewiesenen Nutzens, bei mangelndem politischem Interesse, derzeit schwer durchsetzbar.

## Die Defi-Drohne – die Idee und ihre Umsetzung

Um diese Versorgungslücke zu schließen, ist die Entwicklung von Alternativen zum stationären AED-Netzwerk folgerichtig. Nach der spektakulären Videopräsentation der Designstudie von Mormont von der TU Delft [11] im Jahr 2014 ist der Einsatz von Defi-Drohnen zusehends ins wissenschaftliche und öffentliche Interesse gerückt.

### Mathematische Modelle

Die Idee der Defi-Drohne wurde von mehreren Arbeitsgruppen aufgenommen und weiterentwickelt. Die schwedische „Cardiac-Arrest“-Arbeitsgruppe am Karolinska-Institut demonstrierte erstmals mit einer mathematischen Simulation den möglichen Zeitgewinn durch den Einsatz von Drohnen zum direkten Transport von AED an die Einsatzstelle [9]. Weitere Studien beschreiben ebenfalls in mathematischen Simulationsmodellen signifikant verkürzte Eintreffzeiten der Drohnen im Vergleich zum Rettungsdienst. Dies traf nicht nur für ländliche und suburbane Gebiete [16, 30] sondern auch für Großstädte wie Paris, Vancouver und Toronto zu [6, 14, 26]. In Salt Lake County, USA, könnte der Anteil der OHCA-Patienten, die innerhalb einer Minute mit einem AED erreicht werden, durch den Einsatz von Drohnen von 4,3 auf 92 % gesteigert werden [32]. Die Arbeitsgruppe am Karolinska-Institut modellierte auf der Grundlage historischer OHCA-Daten sogar ein nationales Drohnennetzwerk für Schweden [37].

### Umsetzung in die Praxis – Machbarkeitsstudien

Die praktische Machbarkeit des AED-Drohnenkonzepts wurde bis jetzt vorwiegend im Rahmen von Simulationsstudien untersucht, wobei neben der rein technischen Realisierbarkeit jeweils unterschiedliche operationelle und medizinische Aspekte beleuchtet wurden.

Die erste europäische Simulationsstudie verglich die Eintreffzeiten von AED-Drohnen mit historischen OHCA-Einsatzzeiten des Rettungsdienstes in einer ländlichen Region im Raum Stockholm. Es wurden 18 simulierte Einsätze geflogen, wobei die mediane Eintreffzeit der Drohnen 16 min unter denen des regulären Rettungsdienstes lag [10]. Die Drohnen flogen autonom von der regionalen Rettungswache bis an den Notfallort und konnten dort ohne Probleme landen.

Sanfridsson untersuchte die Interaktionen und Erfahrungen von Senioren im Umgang mit AED-Drohnen. In mehreren Simulationen wurden die Teilnehmer durch die Leitstelle telefonisch zur Reanimation angeleitet und über die anfliegende AED-Drohne informiert. In allen Simulationen konnten Ersthelferdefibrillationen erfolgreich von den Senioren durchgeführt werden. Die Arbeitsgruppe fand keine größeren Sicherheitsprobleme im Umgang mit der Drohne sowie eine hohe Akzeptanz des Verfahrens. Wurde die Reanimation von nur einer Person durchgeführt, waren jedoch längere Unterbrechungen der Herzdruckmassage durch das Holen und Anschließen des Defibrillators zu verzeichnen [34].

Der Einsatz von AED-Drohnen setzt intensive regionale Aufklärungsarbeit sowie eine funktionierende Ersthelferversorgung voraus. Laienhelfer und First Responder benötigen ein Grundverständnis von kardiopulmonaler Reanimation, um ein wirksames Frühdefibrillationsprogramm aufzubauen zu können [38]. Darüber hinaus sind AED nicht intuitiv zu bedienen. Deren Anwendung durch untrainierte Laienhelfer kann zu erheblicher Unterbrechung der kardiopulmonalen Reanimation führen [28], was sich ungünstig auf das Outcome auswirkt.

## Integration der Defi-Drohne in die Rettungskette

**Figure 1:**
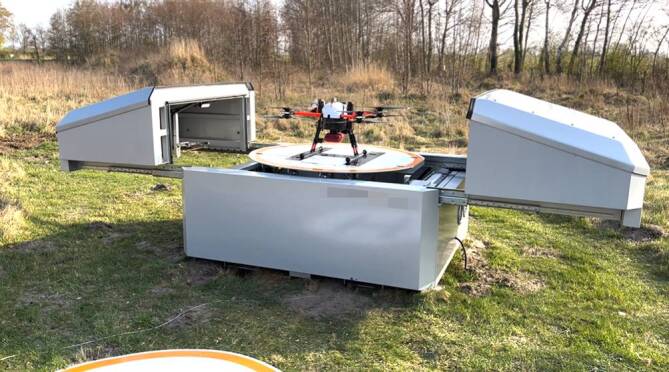

Der nächste Entwicklungsschritt war daher, das AED-Drohnen-Konzept in bestehende Rettungsketten einzubetten. Cheskes [8] konnte in 6 Simulationen die technische Machbarkeit, ein Modell für die Alarmierungskette sowie die erfolgreiche AED-Anwendung durch First Responder (qualifizierte Ersthelfer) demonstrieren. In einer großen Studie der Universität Greifswald konnte in 48 Simulationen an 5 verschiedenen Standorten in Vorpommern nachgewiesen werden, dass Telefonreanimation und die Kombination von AED-Drohnen (Octocopter, Typ „Ceptor“, Fa. Globe UAV, Delbrück, Deutschland, Abb. 1), mit einer gleichzeitigen Alarmierung von First Respondern via Smartphone-App die zeitnahe Defibrillation ohne Unterbrechung der Herzdruckmassage ermöglichen. Bemerkenswerterweise gelang dies auch unter erschwerten Bedingungen wie Regen, hohen Windstärken und eingeschränkten Sichtverhältnissen. Der Umgang mit der AED-Drohne wurde von Beobachtern und Teilnehmern nach definierten Kriterien als sicher und effektiv bewertet. Die befragten Teilnehmer bestätigten eine hohe Akzeptanz von Drohnen in medizinischen Notfällen. Es bestanden einige Unsicherheiten hinsichtlich der Handhabung der AED sowie der rechtlichen Aspekte ihrer Verwendung, die der positiven Gesamtbewertung durch die Anwender jedoch nicht im Wege standen. Die Autoren dokumentierten ebenfalls eine signifikant bessere Reanimationsqualität bei First Respondern als bei Laienhelfern. Diese Ergebnisse legen nahe, den Einsatz von AED-Drohnen mit First-Responder-Systemen zu kombinieren [4]. Dieses Konzept wird im beigefügten Videomaterial illustriert (Zusatzmaterial online: Video 1 und Video 2, s. Box am Anfang des Artikels).

Die erste klinische Erprobung eines AED-Drohnen-Systems fand in Schweden statt. Dem Team gelang der Nachweis, dass AED mit Drohnen auch unter realen Einsatzbedingungen bei Herz-Kreislauf-Stillstand mit Zeitvorteil gegenüber dem Rettungsdienst an die Einsatzstelle gebracht werden können [36]. Im Rahmen dieser Pilotstudie gelang auch die erste erfolgreiche Defibrillation eines 72-jährigen Patienten, der beim Schneeschaufeln einen Herz-Kreislauf-Stillstand erlitten hatte [15].

## Entwicklungsperspektiven

Bis jetzt ist die Defi-Drohne als vielversprechendes Konzept einzuordnen, das an der Schwelle zur klinischen Implementierung steht. Die vorliegenden Studien belegen, dass die Defi-Drohne das Zeitintervall vom Notrufeingang bis zur ersten Defibrillation signifikant verkürzen kann und damit zur einer Verbesserung des Outcomes von OHCA beitragen könnte.

Die Greifswalder Arbeit zeigt, dass die Integration von AED-Drohnen in den deutschen Rettungsdienst prinzipiell möglich ist. Die Akzeptanz der Bevölkerung stellt kein Problem dar, vorausgesetzt, die Implementierung des Systems wird von entsprechender Aufklärungsarbeit begleitet [4].

Grundsätzlich sind Beyond-the-Visual-Line-of-Sight (BVLOS)-Drohnenflüge, die von Behörden und Organisationen mit Sicherheitsfunktionen (BOS) durchgeführt oder angeordnet werden, genehmigungsfrei. Auch Betriebsverbote, die für zivile Betreiber gelten, müssen von BOS-Betreibern nicht beachtet werden. Dennoch sind BOS-Betreiber dazu angehalten, die Vorgaben der europäischen Drohnenverordnung [16] einzuhalten.

Die technischen Voraussetzungen für einen sicheren autonomen BVLOS-Flug sind ebenfalls gegeben. Selbstständig auslösende Notfallschirme bei Antriebsausfall, Antikollisionssysteme, die das Umfliegen von stationären Hindernissen sowie ein Ausweichen vor anderen Flugobjekten erlauben, befinden sich bereits im praktischen Einsatz. Transpondersysteme für Luftverkehrsteilnehmer im unkontrollierten Luftraum (bis 300 m Höhe über Boden), wie FLARM [18], erlauben die frühe Erkennung von anderen Flugkörpern. Solche Systeme sind besonders hilfreich, wenn Drohneneinsätze mit Luftrettungseinsätzen abgestimmt werden müssen. Bedauerlicherweise sind derartige Systeme für den Flugbetrieb im unkontrollierten Luftraum noch nicht vorgeschrieben und werden daher nicht regelhaft angewendet.

Auch der Nachtbetrieb von AED-Drohnen wurde erfolgreich erprobt. Unveröffentlichte Ergebnisse der Arbeitsgruppe „Defi-Drohnen“ am Universitätsklinikum OWL, Campus Bielefeld-Bethel, belegen, dass Nachtflüge mithilfe eines Infrarot-Nachtsichtsystems ohne weiteren Mehraufwand durchführbar sind. Lediglich die Landung an der Einsatzstelle kann im Einzelfall etwas mehr Zeit in Anspruch nehmen.

**Figure 2:**
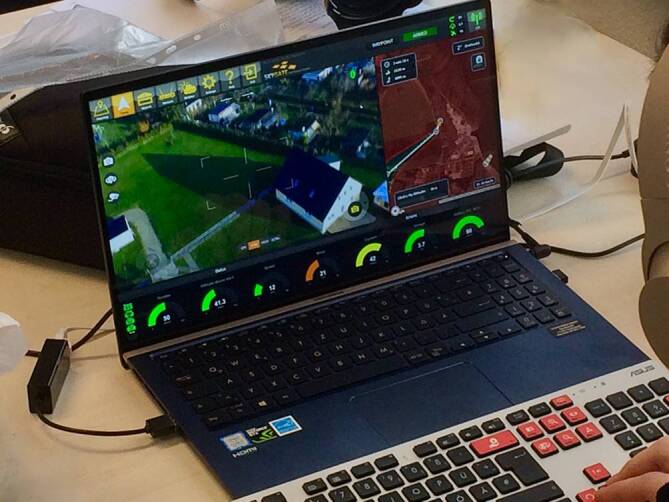

Moderne Steuerungssysteme für Drohnen sind ausgesprochen anwenderfreundlich. Die Benutzeroberflächen sind einfach zu handhaben; zur Aktivierung und zum Start werden nur die Zielkoordinaten der Einsatzstelle benötigt (Abb. 2). Die Einbindung eines Drohnensystems in die rettungsdienstliche Alarmierungskette ist daher mit wenig Mehraufwand zu realisieren und kann an die Alarmierung von Ersthelfern gekoppelt werden [4]. Nach Eingabe der Zielkoordinaten und Überprüfung des durch die Software vorgeschlagenen Flugplans startet die AED-Drohne in der Regel 30 s nach Erteilen des Flugauftrags und fliegt dann ohne weitere Pilotenintervention an die Einsatzstelle [4]. Die Kommunikation mit der Drohne erfolgt über das Mobilfunknetz. Nach Erreichen der Zielkoordinaten sucht der Pilot mithilfe einer hochauflösenden Kamera eine geeignete Stelle aus, an der die Drohne autonom landet und den AED abwirft. Hierbei hat es sich als hilfreich erwiesen, mit First Respondern zusammenzuarbeiten, um die Sicherheit an der Einsatzstelle zu erhöhen und Unterbrechungen der Reanimation zu vermeiden [4]. Alternativ kann eine Drohne auch über der Einsatzstelle schweben und den AED per Winde herablassen [36].

Selbst die notorisch schlechte mobile Netzabdeckung in Deutschland beeinträchtigt die Flugsicherheit kaum: Geht der Kontakt mit dem mobilen Netz verloren, fliegt die Defi-Drohne automatisch die festgelegte Flugstrecke zurück zur Basis. Im Streckenflug, der in einer Flughöhe von 50–120 m erfolgt, herrschen in der Regel sehr gute Netzwerkverbindungen. Wenn überhaupt, treten Verbindungsstörungen in Bodennähe auf. Bemerkenswert ist, dass die teilweise mangelhafte mobile Netzabdeckung in Bodennähe, bei allen 70 von der Greifswalder und der Bielefelder Arbeitsgruppe durchgeführten Einsatzsimulationen, einmalig zu einer leichten Beschädigung der Drohne, aber nie zu Flugabbrüchen geführt hat [4].

Die vorliegenden Arbeiten erlauben den Schluss, dass die moderne Drohnentechnologie den schnellen und sicheren Transport von AED an die Einsatzstelle gewährleistet. Perspektivisch bietet sich für AED-Drohnen eine kostensenkende, multifunktionelle Nutzung an, denn einsatzbereite Defi-Drohnen könnten ebenfalls von einer Rettungsleitstelle, ohne vorherige Umrüstung, zur Erkundung von Schadenslagen, Suchaktionen oder im Zivilschutz eingesetzt werden [39, 42]. Auch der schnelle und wirtschaftliche Transport von Blutprodukten und Laborproben ist ein mögliches Anwendungsgebiet für Drohnen [30, 41].

## Die Kosten-Nutzen-Frage

Die Kosten für ein AED-Drohnen-Netzwerk lassen sich derzeit lediglich abschätzen und hängen von verschiedenen Faktoren ab, die wir kurz darstellen und in einer anderen Arbeit näher beleuchten [33].

- gewünschte *Eintreffzeiten*, die wiederrum die erforderliche Netzwerkdichte und die entsprechende Anzahl der Defi-Drohnen-Standorte bestimmen.
- Regionale *Verfügbarkeit stationärer AED.*
- Regionale *Rettungswachendichte.*
- *Drohnentyp* mit den entsprechenden Anschaffungs- und Unterhaltungskosten: Anschaffungskosten pro Drohne mit Steuerungssystem 30.000–50.000 €, automatischer, beheizbarer Drohnenport: 60.000 €, Wartungskosten: 500–1000 € monatlich; diese Zahlen sind lediglich Schätzungen und basieren auf dem Betrieb eines singulären Drohnensystems (nach Angabe der Fa. Globe UAV); beim Betrieb mehrerer Systeme dürften die Kosten deutlich niedriger ausfallen.
- Die neueste Version des von uns in Bielefeld und Greifswald getesteten Systems erreicht eine *Fluggeschwindigkeit* von 80 kmh^−1^ und deckt somit einen 6 km Radius in 8 Flugminuten ab. Gemittelt müssen 1:35 min von der Alarmierung bis zum Erreichen der Reiseflughöhe sowie 1:00 min zur Landung an der Einsatzstelle hinzugerechnet werden (unveröffentlichte eigene Daten).
- Die Möglichkeit der *gemeinsamen Nutzung* mit anderen Behörden und Organisationen mit Sicherheitsfunktion [42], wie Feuerwehr, Katastrophenschutz und Polizei, würde über die höhere Auslastung einen kostensenkenden Effekt generieren.

Die oben angeführten Einflussgrößen erlauben, zumindest im Ansatz, die Erstellung eines regionalen Kostenmodells.

Aus gesundheitsökonomischer Sicht erscheint der Einsatz von Defi-Drohnen sinnvoll. Eine mathematische Simulation aus Washington, USA, legt nahe, dass die Kombination von First-Responder-Systemen mit AED-Drohnen im Vergleich zu stationären AED-Netzwerken eine signifikante Verbesserung der Überlebensraten nach OHCA kosteneffizient ermöglichen könnte [24]. Zum selben Schluss kommt eine weitere mathematische Simulation, in der ein AED-Drohnen-Netzwerk für den US-Bundesstaat North Carolina entworfen wurde [5]. Der Einsatz von durch Rettungsleistellen kontrollierten Defi-Drohnen könnte im Vergleich zum stationären AED-Netz in Deutschland ebenfalls eine effizientere Variante darstellen, um die Frühdefibrillation zeitnah und flächendeckend anzubieten. [3]: Beim Einsatz von Tragflächendrohnen, die aufgrund höherer Geschwindigkeit eine größere Reichweite erzielen, könnten mit 1074 Drohnenstandorten eine bundesweit 90 %ige Abdeckung von bewohnten, außerstädtischen Gebieten mit längeren Rettungsdienstanfahrzeiten (Zeit Notrufeingang bis zur Defibrillation > 10 min) erreicht werden. Hierdurch könnten im Jahresdurchschnitt 1661 zusätzliche Lebensjahre gewonnen werden. Dies entspricht bei veranschlagten durchschnittlichen jährlichen Systemkosten von 24,2 Mio. € einer Incremental Cost-Effectiveness Ratio von 14.548 € und gilt nach den strengen Kriterien des britischen National Institute for Clinical Excellence als kosteneffektiv [29].

## Herausforderungen

Die technologischen Voraussetzungen für den Einsatz der Defi-Drohnen sind im Großen und Ganzen erfüllt. Dennoch gibt es einige Aspekte, die der weiteren Bearbeitung bedürfen.

- Eine schwedische Simulationsstudie fand kontraproduktive Unterbrechungen der Herzdruckmassage bei allein tätigen Laienhelfern, die durch die Ankunft der Drohne und die Inbetriebnahme des AED verursacht wurden [33, 34]. In der Greifswalder Untersuchung, in der mit First Respondern gearbeitet wurde, traten allerdings keine Unterbrechungen der Herzdruckmassage auf [4] Es stellt sich die Frage, ob der Prozess für Laienhelfer, die allein agieren, optimiert werden kann, oder ob von einem Defi-Drohnen-Einsatz besser abgesehen werden sollte.
- Der Einsatz von senkrechtstartenden Tragflächendrohnen würde die Geschwindigkeit und den operativen Radius von AED-Drohnen-Systemen erheblich erhöhen. Dies wäre kostensenkend, da weniger Drohnensysteme pro km^2^ Fläche erforderlich wären. Allerdings sind Tragflächendrohnen als AED-Drohnen bis jetzt noch nicht zu Anwendung gelangt.
- Die 24-h-Einsetzbarkeit von AED-Drohnen-Systemen müsste gewährleistet sein. Die Ergebnisse der Bielefelder Arbeitsgruppe legen dies zwar nahe, es handelt sich bei dieser Untersuchung allerdings um eine Machbarkeitsstudie, und die Ergebnisse beziehen sich lediglich auf das getestete System (Typ „Ceptor“, Fa. Globe UAV).
- Die Integration in den Arbeitsablauf der Rettungsleitstelle müsste erfolgen. Auch wenn die Alarmierung per Knopfdruck möglich und bis auf eine Flugroutenanpassung keine Intervention bis zum Erreichen der Zielkoordinaten erforderlich ist, müssten Leitstellendisponenten zu Drohnenpiloten ausgebildet werden und im Störungsfall die Steuerung übernehmen zu können.
- Telefonreanimationsprotokolle müssten angepasst und validiert werden, um sicherzustellen, dass durch den Einsatz der Drohne die Reanimation nicht unterbrochen wird.
- In Zusammenarbeit mit First-Responder-Organisationen müssten Ausbildungsprogramme auf die Anwendung von AED und den Umgang mit Drohnen erweitert werden. Auch sollten Defi-Drohnen-Systeme mit existierenden AED-Registern vernetzt werden, um die schnellstmögliche Zuführung eines AED sicherzustellen.
- Transpondersysteme für den Flugverkehr im unkontrollierten Luftraum (unter 300 m Flughöhe) sollten gesetzlich vorgeschrieben werden.

## Fazit

Um das OHCA-Outcome in Deutschland zu verbessern, ist die Anhebung der Ersthelferreanimations- sowie der Frühdefibrillationsraten erforderlich. Während dies bei der Ersthelferreanimation zu gelingen scheint, bleiben die Frühdefibrillationsraten mit unter 2 % in Deutschland zu niedrig. Hier könnte die Defi-Drohne im Rahmen eines Gesamtkonzepts zur Verbesserung der Frühdefibrillation eine Rolle spielen [7]. In Anbetracht der sich schnell entwickelnden Technologie, der vielversprechenden Pilotstudien und der guten Akzeptanz in der Bevölkerung, ist es an der Zeit, den Transfer des Defi-Drohnen-Konzepts in die klinische Praxis anzugehen. Hierzu wären sektorenübergreifende, klinische Feldversuche der konsequente, nächste Schritt.

## Supplementary Information






